# Frequent patient retraining at home reduces the risks of peritoneal dialysis-related infections: A randomised study

**DOI:** 10.1038/s41598-018-30785-z

**Published:** 2018-08-27

**Authors:** Jae Hyun Chang, Jieun Oh, Sue K. Park, Juyeon Lee, Sung Gyun Kim, Soo Jin Kim, Dong Ho Shin, Young-Hwan Hwang, Wookyung Chung, Hyunwook Kim, Kook-Hwan Oh

**Affiliations:** 1 Department of Internal Medicine, Gachon University Gil Medical Center, Gachon University School of Medicine, Namdong Gu, Incheon, 21565 Korea; 2 Department of Internal Medicine, Kangdong Sacred Heart Hospital, Hallym Kidney Research Institute, Hallym University, Kangdong Gu, Seoul, 05355 Korea; 3 Department of Preventive Medicine, Seoul National University College of Medicine, Seoul, Korea; 4 Department of Biomedical Science, Seoul National University College of Medicine, Seoul, Korea; 5 Cancer Research Institute, Seoul National University, Chongno Gu, Seoul, 03080 Korea; 6 Department of Internal Medicine, Hallym University Sacred Heart Hospital, Dongan Gu, Anyang, Gyeonggi-do 14068 Korea; 7 Department of Internal Medicine, Eulji University College of Medicine, Hangeul Biseok Ro, Seoul, 01830 Korea; 8 Department of Internal Medicine, Gachon University Gil Medical Center, Gachon University School of Medicine, Namdong Gu, Incheon, 21565 Korea; 9 Department of Internal Medicine, Yonsei University College of Medicine, Gangnam Severance Hospital, Kangnam Gu, Seoul, 06273 Korea; 10 Department of Internal Medicine, Seoul National University College of Medicine, Seoul, 03080 Korea

## Abstract

The present study, entitled Trial on Education And Clinical outcomes for Home PD patients (TEACH), investigated the effect of frequent retraining at home on the outcomes of peritoneal dialysis (PD). TEACH is a multicentre, open-label, randomised, controlled trial with parallel arms. Patients starting PD were randomized into either the conventional retraining group (CG) or the frequent retraining group (FG). Patients in the FG were given more frequent home visits for retraining. The primary endpoint was exit site infection (ESI). Secondary endpoints were peritonitis, any PD-related infections, hospitalization, technique failure, and patient survival. A generalised estimating equations (GEE) approach was employed for the adjusted effect of training level on the outcomes. Cox regression was employed for peritonitis and other secondary outcomes. The subjects were randomised to either the FG (n = 51) or the CG (n = 53). Although the time of initial training did not differ between the 2 groups, the total time of training was longer and the frequency of training visits was higher in the FG. In the GEE model, the p-values for interactions between groups and time were significant for both ESI and any PD-related infections, suggesting that the event rates of the two groups significantly changed over time. The event rates for the FG decreased over time, and the event rates for the CG increased after month 12. In the older subgroup (age ≥ 60), frequent retraining had a significant effect in the risk reduction of the first episode of peritonitis (adjusted HR 0.01 [0.001–0.35], p = 0.01). Frequent retraining at home reduced the risk of PD-related infections.

## Introduction

Patient training is a fundamental part of a successful peritoneal dialysis (PD) program^1^; moreover, instruction allows patients to achieve adequate self-care, to prevent PD-related infections and, finally, to maintain good health. However, previous studies investigating the effect of patient training for the improvement of PD outcomes are retrospective, observational, or limited by small numbers of participants. One retrospective analysis, which aimed to evaluate the effect of a patient training program on overall survival and technique survival, did not prove its benefit^2^. Another study^3^ with a randomised prospective design evaluated the effect of a structured training program based on adult learning theory. It showed a reduced rate of exit site infection but no difference in the peritonitis rate. To date, no randomised trials have shown that patients with increased training experience reduced risk of peritonitis or improved PD outcomes. Furthermore, controversies still exist regarding patient training strategies such as the optimal duration and frequency of initial training, timing and frequency of retraining, and the sites for PD training.

The present study, entitled Trial on Education And Clinical outcomes for Home PD patients (TEACH), investigated whether frequent patient retraining at home on a regular basis after starting PD can reduce the incidence of PD-related infections and improve patient outcomes.

## Results

### Characteristics of the study population

**Figure 1:**
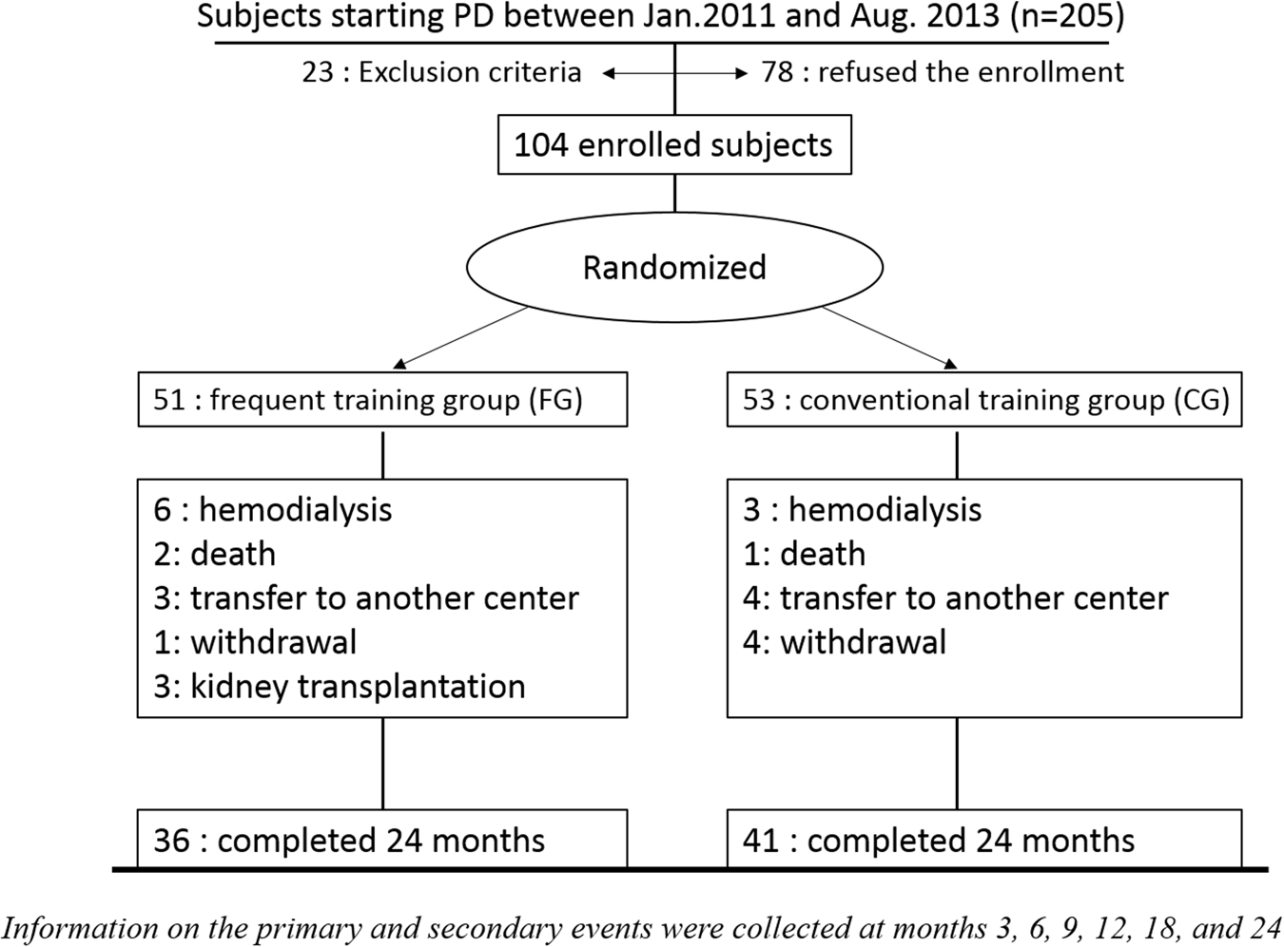
Enrolment status of the study. Among the 205 subjects who started peritoneal dialysis during the study period, 23 subjects were excluded from the study in the screening process (10: previous history of any renal replacement therapy; 4: subjects housed in a nursing home; 4: severe visual disturbance; 5: could not perform PD procedure independently). Seventy eight subjects refused the study enrolment. The remaining subjects (N = 104) were finally enrolled in the study.

Between January, 2011 and August, 2013, 205 subjects started PD in participating centres, among whom 104 subjects were finally enrolled in the study. The patients were randomised into either the frequent retraining group (FG, n = 51) or the conventional retraining group (CG, n = 53). Of the 104 subjects initially enrolled, thirty six (71%) from the FG and forty one (77%) from the CG finished the 24-month study (Fig. 1). The overall drop-out rate and the causes for drop-out were not statistically different between the two groups. At baseline, there were no significant differences (Table 1) in age, diabetes, cause of renal failure, academic years, biochemical parameters and residual renal function (RRF). The CG included more male patients (73.6% vs 54.6%, p = 0.047). Both the FG and CG included a similar number of PD patients receiving treatment from large and small centres.

**Table 1:** Demographic and clinical profiles at baseline in the Trial on Education And Clinical outcomes for Home PD patients (TEACH) study.

	Frequent retraining (FG, N = 51)	Conventional retraining (CG, N = 53)	*P*
	N (%)	N (%)	
Male	28 (54.9)	39 (73.6)	0.05
Age	49.2 ± 11.5	50.7 ± 12.4	0.51
Age ≥ 60	11(22.0)	17 (32.0)	0.27
Insurance			
Medicaid	5 (9.8)	6 (11.3)	0.80
Health insurance	46 (90.2)	47 (88.7)	
Academic year			
≤9 yr	13 (25.5)	12 (22.6)	0.93
9~12 yr	15 (29.4)	18 (34.0)	
>12 yr	23 (45.1)	23 (43.4)	
Past history of DM	31 (60.8)	30 (56.6)	0.66
Cause of ESRD			0.06
Diabetes	29 (56.9)	28 (52.8)	
Hypertension	9 (17.6)	3 (5.7)	
Glomerulonephritis	10 (19.6)	11 (20.8)	
Others	3 (5.9)	11 (20.8)	
Charlson Comorbidity Index			
≤2	16 (31.4)	18 (34.0)	0.78
3~4	23 (45.0)	23 (43.3)	
≥5	12 (23.6)	12 (22.7)	
PD Centre size			
Small: large centre	18:33	17:36	0.84
	**Mean (SD)**	**Mean (SD)**	
BMI (kg/m^2^)	23.6 ± 3.9	23.5 ± 3.8	0.89
SBP (mmHg)	127.7 ± 17.1	130.5 ± 19.5	0.45
DBP (mmHg)	79.2 ± 10.4	79.6 ± 10.8	0.85
Haemoglobin (g/dL)	10.2 ± 1.4	9.8 ± 1.5	0.16
Serum albumin (g/dL)	3.5 ± 0.6	3.5 ± 0.5	0.64
CRP (mg/L)	2.9 ± 6.0	7.1 ± 19.5	0.19
**Dialysis-related parameters at baseline**			
CAPD:APD	47:4	52:1	0.20
D/P cr	0.74 ± 0.19	0.76 ± 0.13	0.57
Total Kt/V	2.14 ± 0.67	1.89 ± 0.69	0.10
nPNA (g/kg/day)	0.99 ± 0.25	0.95 ± 0.24	0.44
RRF (ml/min/1.73 m^2^)	6.93 ± 4.69	5.89 ± 5.31	0.34

Abbreviations: DM, diabetes mellitus; ESRD, end-stage renal disease; BMI, body mass index; SBP, systolic blood pressure; DBP, diastolic blood pressure, CRP, C-reactive protein; CAPD, continuous ambulatory peritoneal dialysis; APD, automated peritoneal dialysis; nPNA, normalized protein nitrogen appearance; RRF, residual renal function estimated by the mean of urea and creatinine clearance calculated from 24 h urine collection. Small centres were defined as those with <100 PD patients and large centres *vice versa*.

### Training

Over the 24-month trial period, subjects in the FG received more frequent training visits (10.6 ± 7.5 days vs 3.6 ± 3.6 days; p < 0.001). The number of unscheduled training visits did not vary between the two groups (1.2 ± 2.0 days vs 0.9 ± 1.9 days, FG vs CG, p = 0.51). The total time spent on PD training was longer in the FG (20.3 ± 9.4 hours vs 11.7 ± 6.7 hours; p < 0.001).

### Exit site infection and any PD-related infection

**Figure 2:**
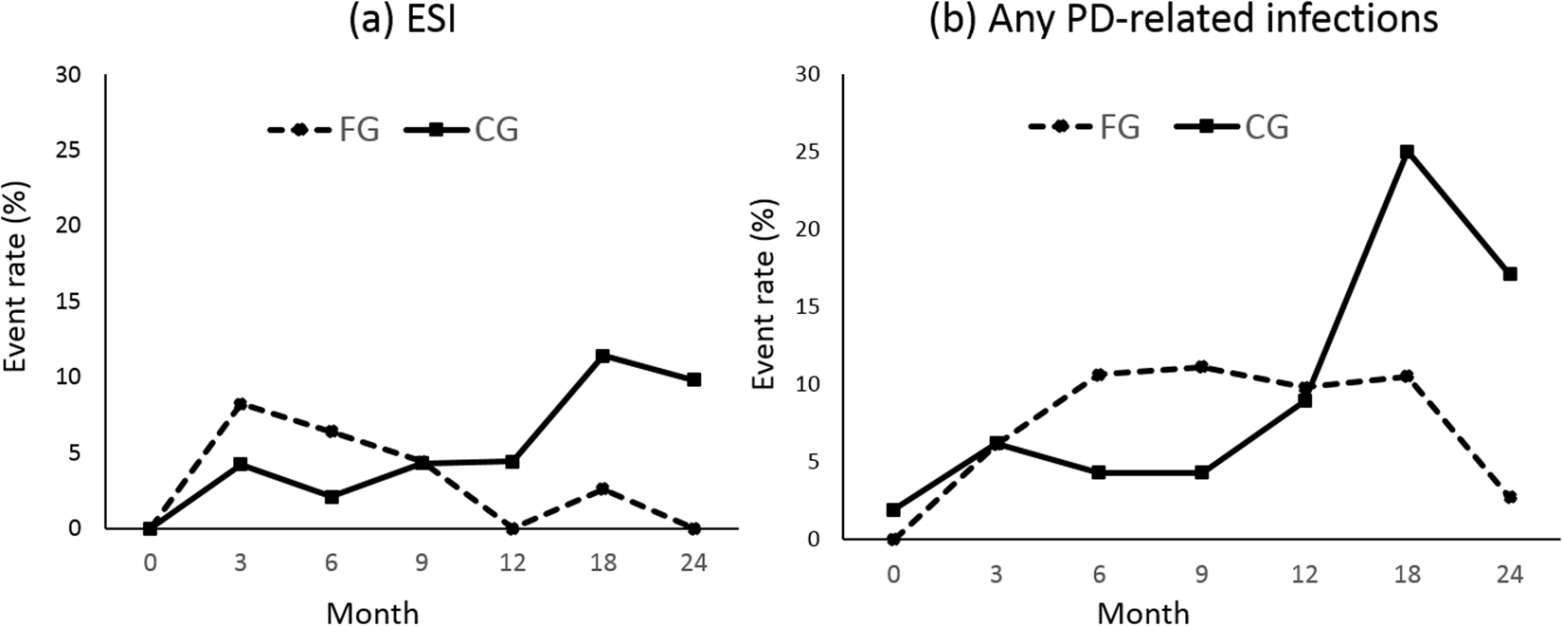
Event rates for exit site infection (**a**) and any PD-related infections (**b**) at each follow-up interval. Abbreviations: FG, frequent training group; CG, conventional training group.

The overall ESI rate for the total study population was 0.17 episode per year at risk (1 episode/72 patient-months). In Table 2, the event rates (ESI and any PD-related infections) for the FG are higher at an earlier period in the study, as compared to the CG. However, this difference is not significant since the p-value for the group difference was >0.05 for both ESI and any PD-related infections in our generalised estimating equations (GEE) model. In the GEE model, the p-values for interactions between groups and time (interaction terms for group x time) were significant for both ESI and any PD-related infections, suggesting that the event rates of the two groups significantly changed over time. As shown in Fig. 2, event rates for the FG decreased over time, and the event rates for the CG increased after month 12.

**Table 2:** The impact of frequent retraining on the ESI and any PD-related infection events, relative to conventional retraining in the Trial on Education And Clinical outcomes for Home PD patients (TEACH) study.

	FG (N = 49)^a^	CG (N = 48)^a^	RR^b^	HR (95% CI)	ER	FG (N = 47)^a^	CG (N = 47)^a^	RR^b^	HR (95% CI)	ER
	ESI events N (%)	ESI events N (%)				Any PD-related infection N (%)	Any PD-related infection N (%)			
Baseline (Time point 0)	0 (0)	0 (0)	NA	NA	NA	0 (0)	1 (1.9)	NA	NA	−1.90
Month 3 (Time point 1)	4 (8.2)	2 (4.2)	1.95	2.00 (0.37–10.93)	4.00	3 (6.1)	3 (6.2)	0.98	0.97 (0.20–4.80)	−0.10
Month 6 (Time point 2)	3 (6.4)	1 (2.1)	3.04	3.07 (0.32–29.51)	4.30	5 (10.6)	2 (4.3)	2.47	2.44 (0.47–12.59)_	6.30
Month 9 (Time point 3)	2 (4.4)	2 (4.3)	1.02	1.05 (0.16–8.14)	0.10	5 (11.1)	2 (4.3)	2.58	2.71 (0.54–14.39)	6.80
Month 12 (Time point 4)	0 (0)	2 (4.4)	NA	NA	−4.40	4 (9.8)	4 (8.9)	1.10	1.19 (0.30–4.77)	0.90
Month 18 (Time point 5)	1 (2.6)	5 (11.4)	0.23	0.24 (0.03–2.07)	−8.80	4 (10.5)	11 (25.0)	0.42	0.46 (0.15–1.44)	−14.50
Month 24 (Time point 6)	0 (0)	4 (9.8)	NA	NA	−9.80	1 (2.7)	7 (17.1)	0.16	0.17 (0.02–1.37)	−14.40
Overall infection rate^d^ (per patient-year)	0.144	0.168				0.216	0.336			
		*P* ^c^					*P* ^c^			
Group (FG vs CG)		0.09					0.20			
Time point		0.56					**0.01**			
Group x Time point		**<0.01**					**0.03**			

Abbreviations: FG, frequent retraining group; CG, conventional retraining group; ESI, exit site infection; PD, peritoneal dialysis; RR, relative risk; ER, excess risk; HR, hazard ratio; NA, non-applicable.

^a^Participants having each infection event within 1 month were excluded from this analysis.

^b^Relative risk (RR) and excess risk (ER) as measure of association were calculated by the equations as follows. RR = ${{ER}}_{{FG}}\div{{ER}}_{{CG}}$; ER = ${{ER}}_{{FG}}-{{ER}}_{{CG}}$.

^c^The p-values for the difference in the event rates between the two groups (p-value for group), the p-values for the different event rates from baseline to last follow-up time point (24 months) (p-value for time point) and those for the group difference by passing follow-up time (p-interaction) were calculated in the GEE (Generalized Estimating Equation) models.

^d^*P* > 0.05 for ESI and any PD-related infection rates, respectively.

### Peritonitis

**Figure 3:**
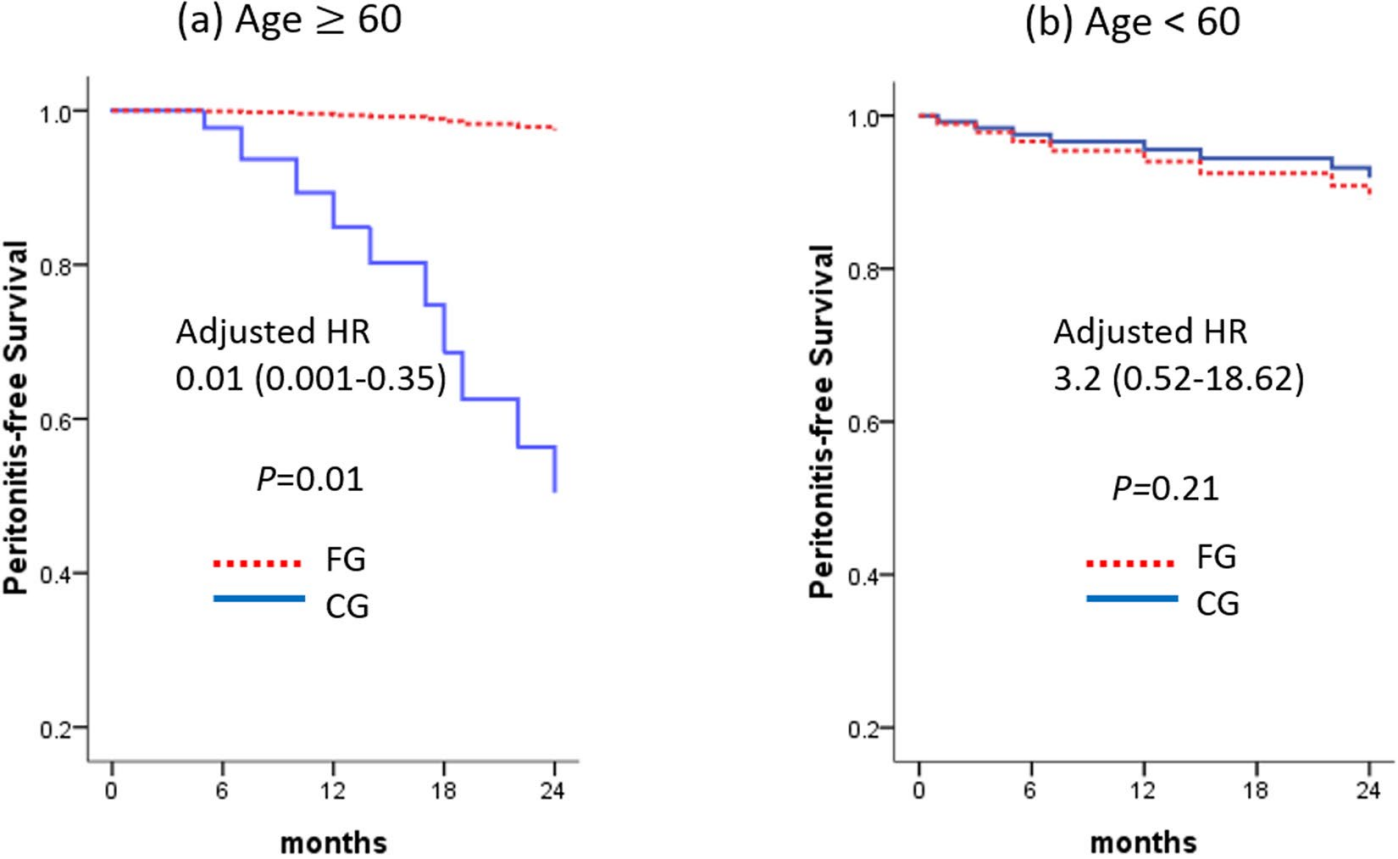
Peritonitis-free survival of the study participants. (**a**) Subgroup of patients with age ≥60, (**b**) subgroup with age <60. In the subgroup with age ≥60, the peritonitis-free survival was significantly lower in the FG compared to the CG (adjusted HR 0.01, *p* = 0.01). Abbreviations: FG, frequent retraining group; CG, conventional retraining group.

The overall peritonitis rate for the total study population was 0.14 episode per year at risk (1 episode/ 86 patient-months). The Cox regression model showed no significant difference between the two groups with regard to the first episode of peritonitis (Table 3). Patient age ≥60 was associated with an increased hazard ratio for peritonitis (Table 3). Therefore, a subgroup analysis for different age groups was made; we stratified the study population into older (age ≥60) and younger (age <60) subgroups. In the older subgroup (Fig. 3a), frequent retraining at home had a significant effect in the risk reduction of the first episode of peritonitis after adjustments for sex, age, DM, centre size, academic year, baseline haemoglobin, albumin and GFR (adjusted HR 0.01 (0.001–0.35), p = 0.01, Fig. 3a). No difference in the risk of peritonitis was noted in the younger subgroup after adjustments (Fig. 3b).

**Table 3:** Hazard ratio (95% confidence interval) for factors associated with the first event of ESI, peritonitis, or any PD-related infections from Cox regression model in the Trial on Education And Clinical outcomes for Home PD patients (TEACH) study.

Variable	ESI			Peritonitis			Any PD-related infection		
	HR^a^	95% CI	*P*	HR^a^	95% CI	P	HR^a^	95% CI	*P*
Training (FG vs CG)	0.76	0.34–1.69	0.50	0.73	0.28–1.87	0.51	0.87	0.45–1.66	0.67
Age group (≥60 vs<60)	1.17	0.52–2.65	0.71	2.60	1.03–6.59	0.04	1.27	0.65–2.46	0.49
PD centre size (large vs small)	0.53	0.24–1.19	0.12	0.92	0.35–2.45	0.87	0.65	0.33–1.26	0.20
	**HR** ^**b**^	**95% CI**	***P***	**HR** ^**b**^	**95% CI**	**P**	**HR** ^**b**^	**95% CI**	***P***
Training (FG vs CG)	0.81	0.35–1.84	0.61	0.70	0.27–1.81	0.46	0.86	0.45–1.66	0.65
Age group (≥60 vs<60)	0.54	0.24–1.22	0.14	2.96	1.16–7.56	0.02	1.37	0.70–2.70	0.35
PD centre size (large vs small)	1.32	0.57–3.06	0.52	0.96	0.36–2.57	0.94	0.67	0.34–1.29	0.23
	**HR** ^**c**^	**95% CI**	***P***	**HR** ^**c**^	**95% CI**	**P**	**HR** ^**c**^	**95% CI**	***P***
Training (FG vs CG)	0.74	0.33–1.68	0.48	0.70	0.36–1.34	0.45	0.52	0.19–1.39	0.19
Education (≥12 vs<12 years)	0.98	0.92–1.05	0.64	0.98	0.93–1.03	0.28	0.97	0.89–1.05	0.40
	**HR** ^**d**^	**95% CI**	***P***	**HR** ^**c**^	**95% CI**	**P**	**HR** ^**d**^	**95% CI**	***P***
Training (FG vs CG)	0.60	0.24–1.50	0.27	0.70	0.27–1.84	0.47	0.70	0.25–1.92	0.49

^a^Crude (unadjusted) hazard ratio in traditional Cox proportional hazard models.

^b^Adjusted hazard ratio in traditional Cox proportional hazard models including age (≥60 vs <60), PD centre size (large vs small), and training group (FG vs CG).

^c^Adjusted hazard ratio in traditional Cox proportional hazard models including education periods (≥12 years vs <12 years) and training group (FG vs CG).

^d^Time-dependent cox proportional hazards regression models.

Abbreviations: ESI, exit site infection; PD, peritoneal dialysis; FG, frequent retraining group; CG, conventional retraining group; HR, hazard ratio; CI, confidence interval; Small centres were defined as those with <100 PD patients and large centres *vice versa*.

### Health-related quality of life (HRQOL), and hospitalization

At the end of the study, quality of life was measured by KDQOL-SF^TM^. The Kidney Disease Component Summary (KDCS) was not significantly different between the FG and the CG groups (62.2 ± 12.9 for the FG, 63.4 ± 13.5 for the CG, p = 0.69). Neither the physical component summary (PCS) nor the mental component summary (MCS) was significantly different between the two groups (for PCS, 55.1 ± 22.4 vs 53.8 ± 22.6, p = 0.81; for MCS, 54.4 ± 21.2 vs. 55.3 ± 21.9, p = 0.85, respectively). No differences were noted in the rate of hospital admission (0.92/patient-year for the FG and 0.94/patient-year for the CG, p = 0.924) and days of hospital admission (5.6 days/patient-year for the FG and 6.9 days/ patient-year for the CG, p = 0.509).

### Patient survival and technique survival

**Figure 4:**
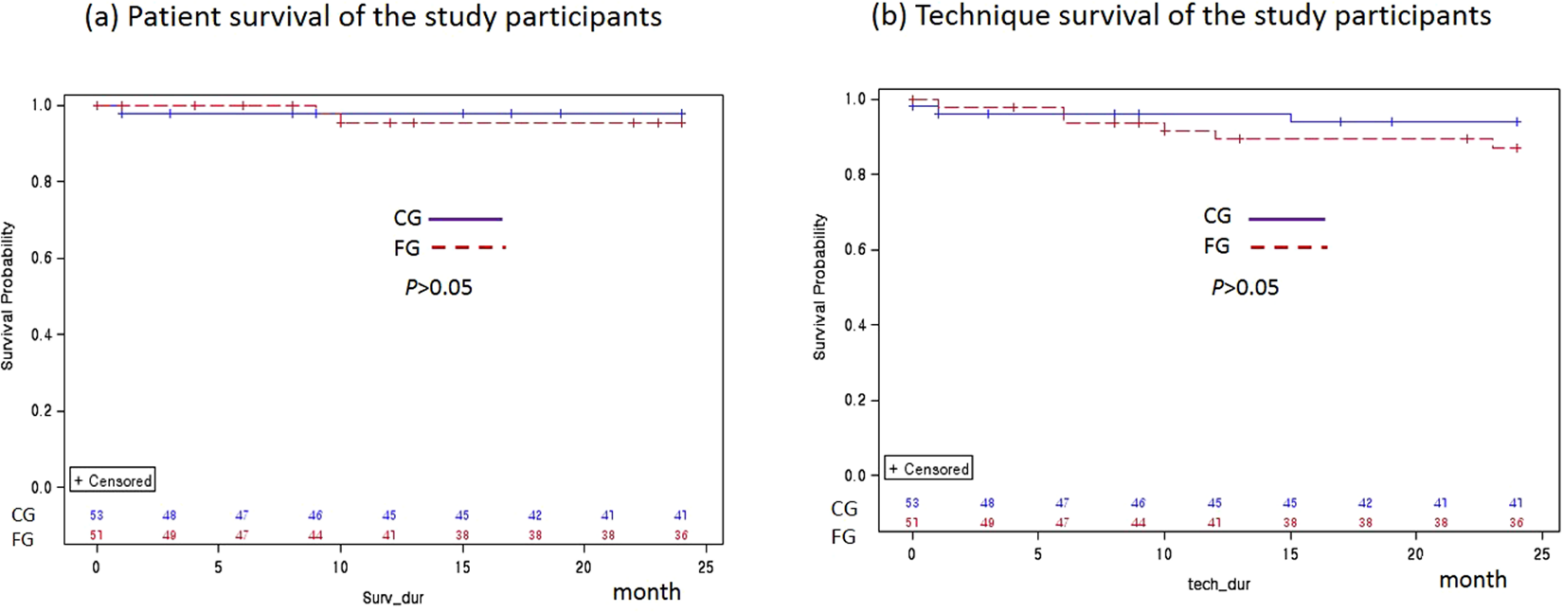
Patient survival (**a**) and technique survival (**b**) of the study participants (*p* > 0.05 by Kaplan-Meier survival analysis). Abbreviations: FG, frequent retraining group; CG, conventional retraining group.

There were three deaths during the study: two from the FG and one from the CG. After adjustments for sex, age, DM, centre size, academic year, baseline haemoglobin, albumin and GFR, there was no difference between the two groups in terms of patient survival (Fig. 4a). Fifteen patients from the FG and twelve from the CG dropped out of the study (Fig. 1). No significant difference was observed in the technique survival after adjustment for sex, age, DM, centre size, academic year, baseline haemoglobin, albumin and GFR (Fig. 4b).

## Discussion

The present study evaluated the impact of frequent patient retraining on the prevention of exit site infection and peritonitis in incident PD patients. The subjects in the frequent retraining group (FG) exhibited significant risk reduction of exit site infection and any PD-related infections over time as compared to the conventional retraining group (CG). Additionally, subgroup analysis revealed that intensive retraining independently reduced the risk of the first episode of peritonitis in older subjects (age ≥60) after adjustments for sex, diabetes, academic year, centre size, haemoglobin and albumin level.

In the present study, the contents and curriculum of the training regime did not differ between the two groups. All patients received the same initial in-centre training during the break-in period. However, the conventional retraining group received only two home visits within two months after starting dialysis, and the frequent retraining group received regular, repeated home visits every one to three months over the entire study period. Therefore, the two groups differed in the number of training visits and total training hours, particularly 2 months after starting PD. The investigators expected that the effect of retraining on the reduction of PD-related infections would manifest after a period of time since only the FG would receive continued retraining over the entire study period. We employed generalised estimating equations (GEE) with binary logistic regression in order to verify this assumption. Table 2 shows that the event rates (ESI and any PD-related infections) for the FG are higher at an earlier period in the study as compared to the CG. However, this difference was not significant; the p-value for the group difference was >0.05 for both ESI and any PD-related infections in our GEE model. In the GEE model, p-values for interactions between groups and time (interaction terms for group x time) were significant for both ESI and any PD-related infections, suggesting that the event rates from the two groups significantly changed over time. As shown in the Fig. 2, event rates for the FG decreased over time, and the event rates for the CG increased after month 12. This could be attributed to the cumulative effect of the frequent and sustained retraining in the FG which showed a reduced risk of ESI or any PD-related infections over time as compared to the conventional retraining group.

Our study subjects were young when compared to another report^3^, and only 27% of the subjects were 60 years or older. The overall peritonitis rates were very low (0.14/year at risk). Therefore, the statistical power was not enough to demonstrate the reduction of peritonitis through frequent repeated training. In our Cox regression analysis, age ≥ 60 was a significant risk factor for peritonitis. Therefore, we performed a subgroup analysis stratified by age ≥ 60 and <60. In the subgroup of patients age ≥ 60, frequent retraining significantly reduced the first episode of peritonitis. These findings suggest that elderly PD patients are exposed to a particularly higher risk of peritonitis, and that the benefit of frequent home visits for repeated training/retraining is maximised in this patient subpopulation^4^.

There have been no randomised trials to determine which location, PD centre or home, is better for patient training. One observational study showed PD patients trained at home had better outcomes than those trained at the PD centre^5^ Another observational study in Italy reported that the peritonitis rate was significantly lower in centres that execute home visits when compared to those that do not^6^. Most of the PD fluid exchanges and self-care procedures are carried out in a patient’s home. Noting that home visits provide information on the way patients function and adapt in their own environments, the ISPD Nursing Liaison Committee strongly recommends home visits for the overall care of PD patients^1^. Further research is needed to determine the timing and frequency of home visits to maximise patient outcomes.

One survey of paediatric PD training showed longer training was associated with lower rates of peritonitis^7^ One study with a large PD cohort evaluated the impact of total training hours on peritonitis and suggested that a minimum of 15 hours is needed to reduce the risk of peritonitis^8^. In the present study, the time spent on training over the 24-month study period was significantly longer for the frequent retraining group (20.3 ± 9.4 hours *vs* 11.7 ± 6.7 hours; p < 0.001), which contributed to the reduction of PD-related infections. We could suggest that over 20 hours of repeated patient retraining over a 24-month period might reduce the risk of ESI and any PD-related infections.

Figueiredo *et al*. observed in a large prospective cohort study that the factors associated with the first episode of peritonitis were education level, duration of training, centre size and timing of training in relation to PD catheter insertion^8^. To date, a few single-centre studies have shown an association between duration of training and lower infectious complications. Some studies addressed an association between training hours and reduced rates of ESI without an impact on peritonitis rates^3,6^. The only studies to have found an association between training hours and lower peritonitis rates were observational or survey in design^7,8^. Only one small single-armed study showed that a multidisciplinary PD education program lowered peritonitis rates^9^. The present randomised, controlled study demonstrated that frequent home training visits, which brought about longer hours of retraining, reduced the incidence of ESI and peritonitis, particularly in the elderly population.

Centre size reflects centre experience in the PD practice, educational system and overall infrastructure for PD. It has been shown that centre size is a significant factor determining the outcomes of PD^10–13^. In the present study, larger centre, arbitrarily defined as those with ≥100 PD patients, exhibited a significantly lower risk of PD-related infections (unadjusted HR 0.51, p = 0.04, Table 3).

Periodic and continued training on a regular basis is crucial. Because patients tend to forget their initial PD training, they may alter the procedures they were taught. One study showed that at the 6th month after starting PD, 51.5% of patients washed their hands improperly and 11.5% forgot to wear a face mask and cap^14^. Sometimes, patients can become complacent about the PD procedure and begin to take shortcuts. An Italian study used a questionnaire to assess patient knowledge about PD and discovered that 34% of patients did not answer the questions correctly^15^. In the present study, subjects in FG received regular retraining visits over the entire study period, which could correct their improper procedures and refresh their memory of PD procedures. Currently, the literature offers no data on the initial and subsequent retraining required after PD initiation. The ISPD Position Statement on the reduction of PD-related infections recommends patient retraining three months after initial training and, thereafter, once yearly at minimum^16^.

In our study subjects, the peritonitis rates (0.084 and 0.156 episodes per patient-year, FG and CG, respectively) were lower than the ESI rates (0.144 and 0.168 episodes per patient-year, FG and CG, respectively). Therefore, our patient population was not large enough to provide the statistical power to determine the effect of frequent retraining on peritonitis. However, ESI is a major risk factor for peritonitis. In particular, catheter-related peritonitis is often refractory and requires PD catheter removal. Therefore, ESI and any PD-related infections were selected as the primary outcomes in our study. Our study showed that frequent and repeated patient training for basic exchange procedures and self-management at home reduced the risk of ESI and overall PD-related infections.

The present study also evaluated the effect of frequent and repeated training on various PD-related outcomes, including hospitalization, quality of life, nutritional status, fluid balance status, technique failure, and patient survival; however, none of the above parameters was statistically significant. This might be due to lack of the statistical power to show a difference in the above parameters. The study subjects were relatively young with low comorbidity and high academic levels, and this must be considered before generalising our findings to the overall PD population.

In conclusion, the present randomised clinical trial has shown that frequent home visits for regular and continued patient retraining reduced ESI and overall PD-related infections. This reduction might be attributed to longer training hours, repeated training over time, home visits, or all of the above.

## Methods

### Study design

The Trial on Education And Clinical outcomes for Home PD patients (TEACH) study aimed to investigate the effect of frequent home visits for repeated training primarily on exit site infection and any PD-related infections in incident peritoneal dialysis (PD) patients. This is a multicentre, open-label, randomised, controlled trial with parallel arms over a 24-month period. (NCT01204619, registered at http://clinicaltrials.gov, date of registration September 17, 2010).

### Study population

This study was performed in six PD centres of university-affiliated hospitals in the metropolitan city of Seoul. A large centre was arbitrarily defined as a PD centre with ≥100 PD patients and a small centre with <100 patients. Approval for the study was obtained from the local ethics committee in six hospitals: Institutional Review Boards of Seoul National University Hospital, Institutional Review Board of Kang Dong Sacred Heart Hospital, Hallym University Sacred Heart Hospital, Gachon University Gil Medical Centre, Eulji General Hospital and Wonkwang University Sanbon Hospital. Patients (age >20 years) who underwent PD catheter insertion for starting PD were enrolled after giving written informed consent. Patients with a previous history of any renal replacement therapy (PD, hemodialysis or kidney transplantation) were excluded from the study. Subjects with severe visual disturbances, dementia, residence in a nursing home, or those who could not perform the PD procedure independently were also excluded.

### Study protocol

Sample size calculation was based on the hypothesis that frequent retraining would reduce the risk of exit site infection by 50% (hazard ratio = 0.5) as compared to the conventional retraining group (α = 0.05, power = 80%). The minimum number of samples was 84. Considering the dropout rate (20%), the target number for enrolment was 104.

After enrolment, the subjects were randomly assigned into either the conventional retraining group (CG) or the frequent retraining group (FG) by sequentially numbered containers, generated by an independent third person. Block-randomization was performed and stratified by the institution and diabetes mellitus. During the two-week break-in period, subjects in both groups received equal, centre-based, one-on-one training taught with the principles of adult learning, a model based on the differences between adults and children and utilises self-actualization in the learning process. The training for FG and CG was provided by the same professional PD nurses in each PD centre. The curriculum was based on the ISPD guidelines and included an overview of PD, aseptic technique, hand washing, exchange procedures, exit site care, diet, and management of complications^1^. After starting PD, both groups were given two equal sessions of training at week 1 and month 2 in their homes by a PD nurse. In addition, the subjects in FG received extra home visits for regular retraining at months 4, 5, 6, 7, 8, 10, 12, 15, 18, 21, and 24. The home training sessions were one hour in length. The content and curriculum of the home training visit were the same for both groups and included basic exchange procedures, fluid balance, infection, diet, medication, and trouble shooting. The PD nurses conducting the training and retraining sessions utilised checklists to assess the patient’s understanding and competency (Supplementary Tables 1, 2, and 3). Any occurrence of an unexpected clinic or home visit for training purposes not pre-specified in the above training plan was recorded as an unscheduled visit.

Information collected at the initiation of the study included age, gender, weight, height, underlying renal disease and comorbidities. Participants had an anthropometric measurement, blood test, peritoneal equilibration test (PET) and Kt/V measurements at regular interval. Health-related quality of life (HRQOL) was assessed using the Korean version of KDQOL-SF^TM^^17–19^ in maintenance PD patients and nutritional assessment was performed using subjective global assessment (SGA) both at months 0 and 24. The modified Charlson comorbidity index was utilised to analyse the patient’s comorbidities^20^. The subjects were evaluated at months 3, 6, 9, 12, 18, and 24 for the primary outcomes, secondary outcomes, and fluid-balance scores. Using a semi-quantitative scale from zero to four, the fluid-balance score was calculated, and patients received 1 point for each of the following: (1) weight within 2 kg of dry body weight; (2) blood pressure <140/90 mmHg; (3) absence of symptoms and signs of volume overload, such as dyspnea, oedema and crackle; and (4) absence of symptoms and signs of dehydration, such as dizziness and orthostatic hypotension^3^. Residual renal function (RRF) was assessed by collecting urine output over a 24-hour period. GFR was calculated as the mean of the values for renal creatinine and urea clearances normalised to 1.73 m^2^ of body surface area (BSA). BSA was calculated by the Du Bois and Du Bois equation^21^.

Dialysis adequacy and protein nutritional status were expressed as (Kt/V)urea, creatinine clearance (L/wk/1.73 m^2^) and normalized protein equivalent of nitrogen appearance (nPNA; g/kg/day). Renal (Kt/V)urea was calculated using data from 24-hour urine collection. Urea distribution volume was calculated using the Watson equation^22^. Dialysate effluent was collected for 24 hours in order to calculate the peritoneal (Kt/V)urea and creatinine clearance (Ccr). Total (Kt/V)urea was calculated by the summation of renal and peritoneal (Kt/V)urea. Modified PET using 3.86% glucose solutions was performed and the peritoneal transport type of each patient was classified as described in detail elsewhere^23^. In short, after overnight dwell with 1.36% glucose PD fluid, patients were subjected to 4-hour dwell with 3.86% glucose dialysis fluid. Blood samples were taken at 2 hours. The dialysate-to-plasma ratios for creatinine at 4 hours (D/Pcr) were calculated as the dialysate concentration at 4 hours divided by the plasma concentration for creatinine. The D/Pcr was corrected for glucose interference using correction factor derived by each laboratory.

Any event including the development of ESI, peritonitis, hospitalization of any cause, cardiovascular event, and death was recorded. A PD-related infection was defined as either ESI, tunnel infection or peritonitis. The study’s primary endpoint, monitored over the entire trial period, was exit site infection (ESI). Secondary endpoints recorded over the entire trial period included peritonitis, any PD-related infections, death, transplantation, cardiovascular events, and technique failure (defined as death or transfer to hemodialysis; censored for transplantation or transfer to another PD unit). Secondary endpoints measured at the end of the trial included health-related quality of life (HRQOL), nutritional status, and fluid status score. The diagnosis and treatment of ESI, tunnel infection and peritonitis were made in accordance with the ISPD guidelines^24,25^. The implementation of the clinical trial was performed in accordance with the Declaration of Helsinki and other relevant guidelines/ regulations.

### Statistical analysis

Survival curves were calculated according to the development of primary and secondary endpoints, and they were compared between the two training groups using the Cox proportional hazard model using SPSS Statistics version 22. Adjustments were made for sex, age, DM, centre size, academic year, baseline haemoglobin, albumin and GFR.

For repeated measures data, Generalised Estimating Equations (GEE) were used to assess the effect of frequent home retraining visits relative to conventional retraining for the primary outcome over 24 months. The patient’s ESI status was dichotomised as ESI (code = 1) vs no-ESI (code = 0) at each time point (months 3, 6, 9, 12, 18 and 24). It was assumed to have a binomial distribution and a logit link was analysed in the model. The GEE model included the fixed effect term (two training groups and time point) and the interaction term between the training groups and time point.

Statistical analysis was performed with SAS version 9.4. (SAS Institute Inc., Cary, NC, U.S.A). A *p*-value < 0.05 was regarded to be statistically significant for all analyses.

The datasets generated and/or analysed during the current study are available from the corresponding author upon reasonable request.

## Electronic supplementary material

Supplementary Information
