# Supplementary material for: Frequent patient retraining at home reduces the risks of peritoneal dialysis-related infections: A randomised study
Source: Sci Rep. 2018 Aug 27;8:12919. doi: 10.1038/s41598-018-30785-z (PMC6110747; doi:10.1038/s41598-018-30785-z)

Frequent patient retraining at home reduces the risks of peritoneal dialysis-related infections : A randomised study

[1]Jae Hyun Chang, M.D., Ph.D

[2]Jieun Oh, M.D., Ph.D

[3]Sue K. Park, M.D., Ph.D

[3]Juyeon Lee, M.S.

[4]Sung Gyun Kim, M.D., Ph.D

[2]Soo Jin Kim, M.D.

[2]Dong Ho Shin, M.D.

[5]Young-Hwan Hwang, M.D., Ph.D

[6]Wookyung Chung, M.D., Ph.D

[7]Hyunwook Kim , M.D., Ph.D

[8]Kook-Hwan Oh, M.D., Ph.D.

Representing **T**rial on **E**ducation **A**nd **C**linical outcomes for **H**ome PD patients **(TEACH) Investigators**

Corresponding Author ;
Kook-Hwan Oh, MD, PhD.
Department of Internal Medicine, Seoul National University College of Medicine,
Chongno Gu, Seoul, 03080, Korea
Tel: +82-2-2072-0776, Fax: +82-2-741-4876
Email: ohchris@hanmail.net

JHC and JO equally contributed to this work.

[1]Department of Internal Medicine, Gachon University Gil Medical Center, Gachon University School of Medicine, Namdong Gu, Incheon, 21565, Korea

[2]Department of Internal Medicine, Kangdong Sacred Heart Hospital, Hallym Kidney Research Institute, Hallym University, Kangdong Gu, Seoul, 05355, Korea

[3]Department of Preventive Medicine, Seoul National University College of Medicine, Seoul, Korea; Department of biomedical Science, Seoul National University College of Medicine, Seoul, Korea; Cancer Research Institute, Seoul National University, Chongno Gu, Seoul, 03080, Korea

[4]Department of Internal Medicine, Hallym University Sacred Heart Hospital, Dongan Gu, Anyang, Gyeonggi-do, 14068, Korea

[5]Department of Internal Medicine, Eulji University College of Medicine, Hangeul Biseok Ro, Seoul, 01830, Korea.

[6]Department of Internal Medicine, Gachon University Gil Medical Center, Gachon University School of Medicine, Namdong Gu, Incheon, 21565, Korea

[7]Department of Internal Medicine, Yonsei University College of Medicine, Gangnam Severance Hospitial, Kangnam Gu, Seoul, 06273, Korea

[8]Department of Internal Medicine, Seoul National University College of Medicine, Seoul, Korea

Supplementary Table 1. Contents for patient education and training

1. What does the kidney do in our body?	• Location of the kidney
	• Functions of the kidney
	• What if the kidney stops working
2. What is peritoneal dialysis?	• What is PD?
	• PD catheter insertion
	• How is the water and waste removed?
	• Types of PD
3. Care of PD	•How PD fluid is exchanged?
	• PD fluid exchange procedure
	• Exit care
	• Shower
	• Water balance
4. Infectious complication	• Peritonitis
	• Exit site infection
5. Diet	• Protein
	• Carbohydrate
	• P
	• K
	• Na
	• Water
6. Drugs	• anti-hypertensive drugs
	• P binder
	• Vitamin D
	• Iron agents

	• Laxatives
	• Erythropoietin and other ESAs
	• Insulin (in case of diabetes)
	• Heparin
	• Antibiotics
7. Trouble shooting	1) Infusion/drain problems
	2) Contamination during exchange
	3) Turbid effluent
	4) Fibrin in effluent
	5) Bloody effluent
	6) Dark yellow effluent
	7) Catheter leakage
	8) Leakage from PD fluid bag
	9) Peri-catheter leak
	10) Abdominal pain
	11) Shoulder pain
	12) Exit pain
8. PD and life	• Daily life
	• Sexual life
	• Exercise
	• Travel
9. PD fluid management	• PD fluid prescription
	• PD fluid delivery

Supplementary Table 2. Checklist for PD training (for the first home visit)

Title	Content	Reference
Understanding of PD	-Kidney function and ESRD<br>-PD vs. HD<br>-Principles of PD<br>-Exchange method with demonstration (aseptic technique, PD environment)<br>-Record the PD diary<br>-Fluid balance<br>-Diet & Exercise<br>- Problem solving<br>- Exit care and shower<br>-T-set exchange period<br>- Medication	PD education manual
PD price	-PD solution price<br>-payment method	
Delivery	-Domestic delivery<br>-No refund, exchange and delivery without prescription<br>-collection of waste bag	
Benefit	-Handicapped<br>-Support from government/National pension	
Before going home	-prepare the supply<br>-delivery the solution<br>-patient test	

Supplementary Table 3. Checklist for retraining during home visits

Patient name:		
Date:		
If retraining is the result of a peritonitis episode, what do you think was the cause?:		
**Hand Washing**	Yes	No
Hand washing technique is adequate<br>Soap is adequate<br>Washing time is adequate<br>Good technique for drying hands<br>No decantation of liquid soap		
**Exchange Technique**	Yes	No
The patient does an exchange:<br>In a suitable room<br>Around animals<br>Checks date, volume, leak in the bag<br>Disinfects the work surface correctly<br>Mask<br>Does the connection the right way<br>“Flush before Fill” is done according to the technique<br>The date of the MiniCap is observed<br>Does the disconnection the right way<br>Reconnects to the MiniCap the right way		
**Cycler technique**	Yes	No
The patient:<br>In a suitable room<br>Around animals<br>Checks date, volume, leak in the bag<br>Is very familiar with his or her programming<br>Is able to change the programming<br>Mask<br>Connects the bags and tubing correctly<br>Does the priming the right way<br>Does the connection the right way<br>Does the disconnection the right way<br>Reconnects to the MiniCap the right way		
**I know what I have to do if:**	Yes	No
I see that my fluid is cloudy<br>I am constipated<br>I am bloated<br>I am dehydrated or tired<br>I contaminate my transfer line<br>I have fibrin in my drain fluid		
**I must do every day**	Yes	No
Weigh myself<br>Take my blood pressure<br>Take note of my general condition (swelling, etc.)<br>Observe my drain fluid		
Comments:		

CONSORT

TRANSPARENT REPORTING of TRIALS

CONSORT 2010 Flow Diagram

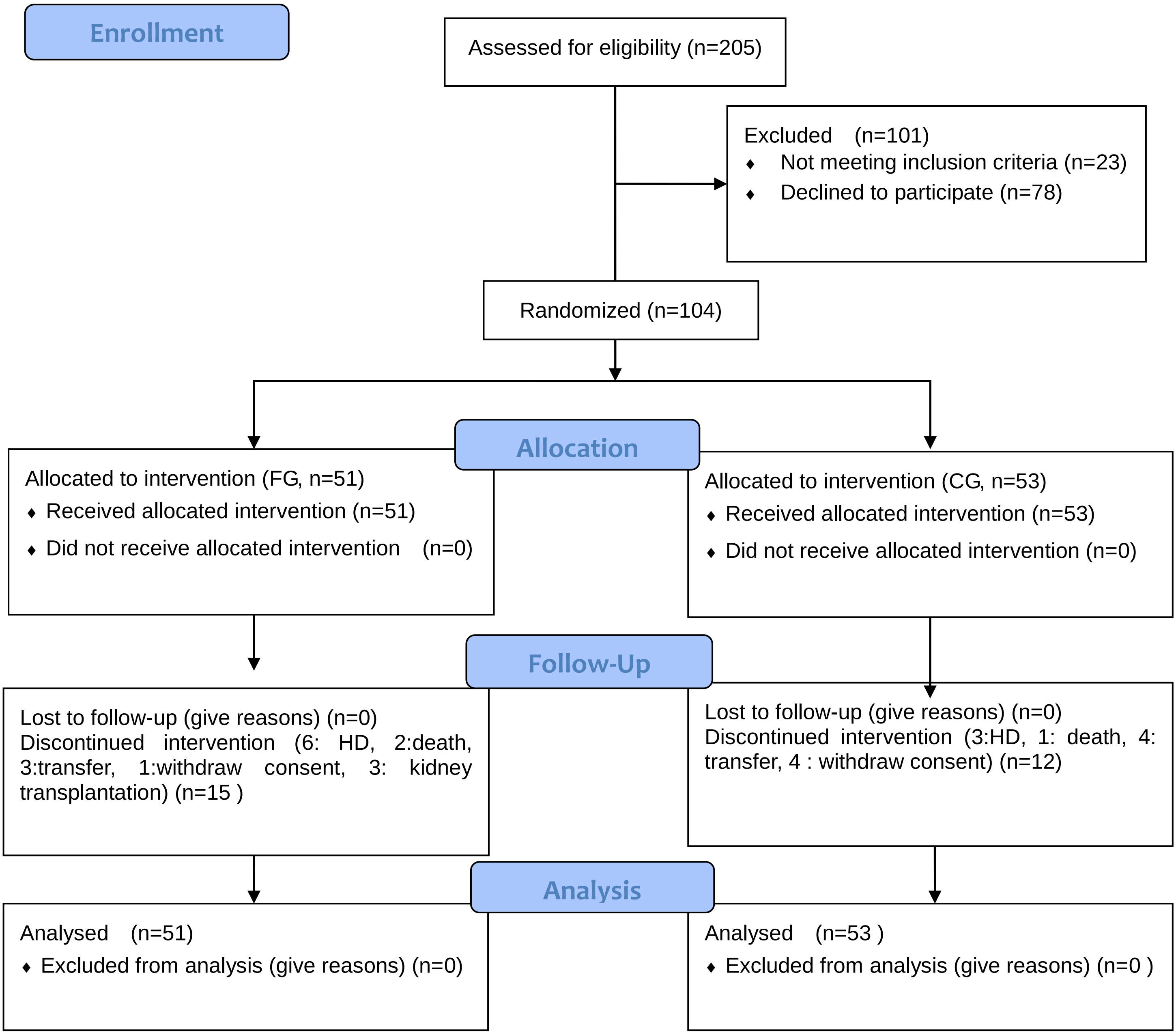

Supplement: Supplementary file 1 — Supplementary Information [file 41598_2018_30785_MOESM1_ESM.pdf]
